# Voluntary Medical Male Circumcision: A Qualitative Study Exploring the Challenges of Costing Demand Creation in Eastern and Southern Africa

**DOI:** 10.1371/journal.pone.0027562

**Published:** 2011-11-29

**Authors:** Jane T. Bertrand, Emmanuel Njeuhmeli, Steven Forsythe, Sarah K. Mattison, Hally Mahler, Catherine A. Hankins

**Affiliations:** 1 Tulane School of Public Health and Tropical Medicine, New Orleans, Louisiana, United States of America; 2 United States Agency for International Development, Washington, District of Columbia, United States of America; 3 Futures Institute, Glastonbury, Connecticut, United States of America; 4 Jhpiego, Dar es Salaam, Tanzania; 5 Joint United Nations Programme on HIV/AIDS, Geneva, Switzerland; Centers for Disease Control and Prevention, United States of America

## Abstract

**Background:**

This paper proposes an approach to estimating the costs of demand creation for voluntary medical male circumcision (VMMC) scale-up in 13 countries of eastern and southern Africa. It addresses two key questions: (1) what are the elements of a standardized package for demand creation? And (2) what challenges exist and must be taken into account in estimating the costs of demand creation?

**Methods and Findings:**

We conducted a key informant study on VMMC demand creation using purposive sampling to recruit seven people who provide technical assistance to government programs and manage budgets for VMMC demand creation. Key informants provided their views on the important elements of VMMC demand creation and the most effective funding allocations across different types of communication approaches (e.g., mass media, small media, outreach/mobilization). The key finding was the wide range of views, suggesting that a standard package of core demand creation elements would not be universally applicable. This underscored the importance of tailoring demand creation strategies and estimates to specific country contexts before estimating costs. The key informant interviews, supplemented by the researchers' field experience, identified these issues to be addressed in future costing exercises: variations in the cost of VMMC demand creation activities by country and program, decisions about the quality and comprehensiveness of programming, and lack of data on critical elements needed to “trigger the decision” among eligible men.

**Conclusions:**

Based on this study's findings, we propose a seven-step methodological approach to estimate the cost of VMMC scale-up in a priority country, based on our key assumptions. However, further work is needed to better understand core components of a demand creation package and how to cost them. Notwithstanding the methodological challenges, estimating the cost of demand creation remains an essential element in deriving estimates of the total costs for VMMC scale-up in eastern and southern Africa.

## Introduction

By 2007, three clinical trials had provided compelling evidence that voluntary medical male circumcision (VMMC) reduces female-to-male HIV transmission by about 60% [1]–[3]. Following a review of these results in 2007, the World Health Organization and the Joint United Nations Programme on HIV/AIDS (UNAIDS) recommended that VMMC services be expanded as part of a comprehensive HIV prevention package, especially in settings with high HIV prevalence, low levels of male circumcision (MC), and generalized heterosexual epidemics [4]. With the support of the international donor community, the following 13 country governments in eastern and southern Africa considered scaling up VMMC services: Botswana, Kenya, Lesotho, Malawi, Mozambique, Namibia, Rwanda, South Africa, Swaziland, Tanzania, Uganda, Zambia, and Zimbabwe.

VMMC service implementation and scale-up success depend on both the supply of, and the demand for, high-quality safe services for those who would most benefit. Elements of supply include voluntary HIV testing and counseling offered prior to the surgery, MC counseling, the surgical procedure; routine follow-up visits and other contacts (e.g., short message service [SMS] messaging to facilitate return in case of adverse events), and emergency services in case of complications. Facility-level costs associated with the supply of VMMC services include direct and indirect labor, material, equipment, and overhead (including maintenance and utilities). Additional costs include those for training, policy development, and supply chain management (including waste disposal). In collaboration, the United States Agency for International Development (USAID) and UNAIDS have assisted countries in estimating the direct costs of scaling up VMMC services by supporting facility-based cost data collection to populate the Decision Makers' Program Planning Tool, which estimates both the cost and HIV impact of various programming options [5], [6]. Such costing studies have been conducted in Kenya, Namibia, South Africa, Uganda, Zambia, and Zimbabwe, and are underway in Tanzania and Malawi.

“Demand” refers to the level of interest or motivation to access services and obtain the procedure on the part of the population prioritized for VMMC. Demand creation aims to influence the attitudes, intentions, and, ultimately, the decisions of men to seek VMMC services. Demand creation comprises a constellation of activities designed to increase awareness of VMMC among both men and women (including guardians of adolescents), provide factual information regarding its benefits, inform the population about where services can be obtained, dispel myths, influence community norms regarding MC, and encourage men and adolescent boys to seek these services, thus increasing the flow of clients to them. We are unaware of any attempts to date to understand and estimate the cost of demand creation for VMMC [7]–[10].

There is a growing literature of acceptability studies that attempt to measure how receptive men in different countries are to MC, the reasons for their interest, and the barriers to getting circumcised [11]–[16]. Westercamp et al. [15], summarizing 13 qualitative studies conducted in nine countries in sub-Saharan Africa, found that 65% of men (range, 29%–87%) were willing to become circumcised, 69% (47%–79%) of women favored circumcision for their partners, and 71% (50%–90%) of men and 81% (70%–90%) of women were willing to circumcise their sons. Whereas acceptability studies provide useful insights into the attitudes, perceptions, and values of men and women in different countries, specific factors that influence the demand for VMMC likely vary by setting, requiring local information gathering to inform demand creation programs.

The aim of this paper is to propose an approach for estimating the costs of demand creation for VMMC scale-up by addressing the following questions. (1) What are the elements of a standardized package for demand creation? (2) What methodological challenges exist and must be taken into account in estimating the costs of demand creation? (3) Taking these challenges into account, what are the recommended steps in costing VMMC demand creation? Drawing on the principles and experience of demand creation in other areas of public health, we explore adaptations to the field realities of VMMC programs in eastern and southern Africa.

## Methods

In any type of multi-country costing exercise, it is important to develop a standard list of items to be costed, even if these countries do not incur all of the expenses and/or incur other costs not on the list. As no such standard package appears to exist for VMMC demand creation, we conducted a key informant study aimed at identifying (1) elements in a standard demand creation package and (2) the percentage of the demand creation budget to allocate to different types of communication (i.e., channel mix).

Using purposive sampling, we recruited seven key informants to participate in the study. We conducted interviews by telephone in early 2011; these lasted an average of 1 h. We obtained written responses when phone conversations were not possible. The discussion guide appears in Text S1. The informants were communication specialists working for agencies providing demand creation technical assistance to African governments actively involved in VMMC scale-up and administering specific budgets for demand creation. The agencies for whom they were working were the Academy for Educational Development/C-Change in Kenya; the Center for Communication Programs, Johns Hopkins Bloomberg School of Public Health in South Africa; Jhpiego, an affiliate of Johns Hopkins University in Tanzania; and Population Services International in Kenya and Zambia.

## Results

### Demand Creation Packages and Channel Mix for Resource Allocation

With respect to the potential elements of a demand creation package and recommended resource allocation across communication channels (i.e., channel mix), key informants emphasized the difficulty of employing a “one size fits all” approach to demand creation, given the spectrum of media environments ranging from urban South Africa to rural Tanzania. Nonetheless, they addressed the question: “What do you consider to be the ideal resource allocation for communication for male circumcision?” Their responses were divided among mass media, print materials (small media), and outreach/mobilization. The recommended budget allocation for mass media relative to all forms of communication channels ranged from 20% to 60% (with responses almost evenly distributed across this range). Most wanted some, if not all, mass media funding to go to radio, with the exception of South Africa, where more than 80% of the population watches television. There was strong support for the use of “targeted radio”―i.e., regional or community stations near VMMC sites that broadcast specific information related to the services provided in that area (e.g., the date and time when services will be available at a particular location). One informant strongly advocated local radio but recommended a relatively small allocation because “local radio is so inexpensive in our country.”

Television, which was allocated less than 10% in all budgets, was not considered essential for several reasons. One informant mentioned that it was useful for creating awareness about the benefits of VMMC at the start of the scale-up but was not an effective mechanism for prompting men to make a decision to undergo VMMC or go to a VMMC site. This same informant would consider using television in a similar situation if the budget for VMMC communication was generous. Other limitations of television that were mentioned were its inability to reach some rural areas and/or the frequent shut-downs of electricity. By contrast, another informant was far more positive about the potential of television, especially drama series, for social modeling (i.e., exposing men to desirable models of behavior, including VMMC). He noted that television drama on its own would be insufficient and that additional advertising would be required, particularly in environments where there is a diversity of media available to consumers. Others commented that television can be useful and inexpensive if a news program does a feature on the VMMC program or a television talk show invites staff to serve as guests. This television coverage comes at no expense to the VMMC program and increases its visibility.

There was little enthusiasm for billboards; only one informant would give them 10% of the budget (for use in urban areas only) and the others would allocate zero. The perceived limitation is that billboards do not lend themselves to communicating a lot of information on a complex topic. Moreover, they generally are not available in rural areas. One informant cited wall hangings outside of shops as a viable alternative to billboards in rural areas. Programs that opt to use billboards can increase their effectiveness by including a link to service delivery, such as a helpline number.

Informants allocated the remaining support (ranging from 30% to 60%) to outreach. Several informants included the costs of print materials (small media) in outreach, presumably because they are an essential element contributing to effective outreach. Programs often had at least two types of brochures: one for the prospective client and one for the female partner. Two informants cited a third type of brochure: in one case, it was for adolescents and their guardians, and in another, a flyer for clients to read at VMMC sites while waiting for the procedure. Other print materials included posters—for use in service delivery locations to announce the next date or ongoing availability of service—and flyers. One program in South Africa had discovered that the cost of small media can be prohibitive, depending on the size of the intended population.

SMS, a form of text messaging, was mentioned by three key informants but none of the respondents included it in the hypothetical allocation of funds across channels. The experience in the Iringa Region of Tanzania, illustrated a potentially effective use of this medium. Although the Iringa Region is rural, many clients and prospective clients own cell phones. At a very low cost, the VMMC program has worked with the local phone provider to make a series of text messages available to the public. If a person texts “TOHARA” (“circumcision” in Swahili), he/she receives two messages on the benefits of VMMC. Texting “WAPI” (“where” in Swahili) sends a message on where and at what times VMMC services will be available. For clients who are circumcised, texting “BAADA” (“after” in Swahili) triggers 8–10 messages over a 6-wk period, including messages on wound care, healing, and abstinence. At 6 wk, men receive a message that it is now permissible to have sex, but that they should use condoms and stick with one partner. These messages are free of charge to the user. The phone company reported that the VMMC program had the largest number of users of any “company” that year. The key informant from South Africa referred to the “combination approach” of alerting cell phone owners to information on a website (which they then would access through their cell phone) and using the messaging service to deliver follow-up reminders for men who opt to be circumcised. He stated that the use of cell phone technology for the promotion of HIV prevention and VMMC needs to be integrated into social mobilization activities through which phone numbers can be gathered, because sending unsolicited SMS messages generally is illegal.

### Key Considerations in Designing and Costing Successful Demand Creation Programs

Key informants provided other useful insights for the design and costing of successful demand creation programs based on their experiences with VMMC demand creation.

- Interpersonal communication is essential for demand creation for VMMC (e.g., community health workers, community mobilizers, small media [brochures]). Mass media can create awareness of VMMC, but interpersonal communication serves as the catalyst to action.
- Programs should take advantage of existing community organizations and partner programs, piggybacking the VMMC content onto the existing outreach efforts of trusted organizations. Efficient use of resources requires training existing outreach personnel to discuss VMMC in the broader context of HIV prevention and paying them when they give talks on VMMC.
- Targeting educational institutions (e.g., schools, universities) allows programs to reach adolescents and young men in these settings–the age groups that to date have proven most receptive to VMMC.
- Integrating VMMC messages into the broader context of HIV prevention (and not as an isolated topic) is essential. The messages must convey that VMMC is only partially protective against HIV transmission and that behavioral risk-reduction strategies remain essential to safer sex strategies to protect oneself and one's sexual partners (e.g., correct and consistent condom use, reducing one's number of partners). Messaging may be intensified during campaign periods, especially to alert the population to dates and locations where teams will be performing VMMC.
- Creating demand for VMMC among men over the age of 25 has proven more difficult in some settings than attracting adolescents and younger men to VMMC services. In estimating future costs, it is essential to take into account the additional costs of strategies tailored to reach this “harder to reach” segment of the population.
- Seasonal variability is also a factor. This type of variability takes two forms: (1) natural, caused by locally occurring events independent of VMMC programs (e.g., school holiday periods, cooler temperatures), and (2) programmatically induced, resulting from efforts by governments to intensify the delivery of services in hopes of stimulating demand during certain periods. In some cases, the intensified programs are scheduled to occur with naturally occurring events (e.g., school holidays). The Rapid Results Initiative in Kenya illustrates this case.
- Collaboration between partners (government, technical assistance agencies, and local community organizations) is essential for ensuring both initial success and sustained effects of VMMC demand creation efforts. Currently, the nature and extent of this collaboration varies by country (e.g., high in Kenya and Tanzania, lower in Zambia), but collaboration remains an important factor in most programs.
- Collaboration also extends to the divide between VMMC and traditional MC. A key informant based on South Africa shed light on the importance of collaboration, although admitted it was still a work in progress. The informant relayed that there was an existing partnership in the Eastern Cape where trained medical professionals aided in traditional MC when medical emergencies necessitated it. Further communication between the two sectors is underway, but according to the informant, other activities have taken priority.
- Rather than individual program branding, a strong national VMMC symbol, such as a logo that links the media messages to actual VMMC service delivery sites, would facilitate personal action by men who have been motivated to seek services.

### Challenges in Estimating the Cost of Demand Creation to Reach Universal Coverage

Given the lack of a standard package of VMMC demand creation activities, country programs have opted to use different channels to reach prospective clients, with a strong focus on interpersonal communication strategies using mobilizers or peer educators. Table S1 outlines the types of mass and interpersonal channels used to date. The lack of a standard or recommended package of communication activities to serve as a starting point for costing demand creation activities for VMMC is by no means unique. In virtually every health domain, effective communication programs aimed at creating demand are those tailored to the specific behavior change or demand creation objective, taking into account context-specific factors.

Even with a standard package of VMMC demand creation activities, costs would vary by country or program, based on factors such as geographical area to be covered, size of the population within the target area, mix of communication channels to be used, number of languages required to reach the majority of the intended audience, readiness of men in a specific community to accept VMMC services (especially regarding the procedure being performed in a clinical setting), availability of in-country production facilities for broadcast or print materials, and cost of media time for broadcast of radio and television spots during peak hours.

Different regions of a country may have contrasting requirements for creating sufficient demand for VMMC services. In Tanzania, for example, current MC prevalence based on self-reporting varies by setting, with urban areas estimated at 88% prevalence and rural areas at closer to 60% [17]. MC prevalence also varies by region, reaching a high of nearly 100% in Lindi and a low of 21% in Shinyanga. As a result of this wide variation, USAID and UNAIDS currently are working with the government of Tanzania and with Jhpiego to evaluate potential demand creation resource requirements for significant scale-up of VMMC services in the eight regions of the country prioritized by the Tanzanian Ministry of Health. This exercise involves evaluating the scale-up required for VMMC services in each region, identifying the most effective communication strategies for those regions, and costing the demand creation strategy.

The cost of demand creation varies both by the comprehensiveness of the communication program and the extent of adherence to standards of best practice for demand creation. Programs that use multiple and reinforcing channels have stronger effects than single channel programs [18], and those that run for a longer duration show greater effects than shorter ones [19]. The norms of best practice dictate the use of formative research and pretesting of materials before production. Costing studies often exclude research costs and focus instead on programmatic costs, but formative research and pretesting are essential parts of the design and implementation of sound communication programs, regardless of the objective. Comprehensive communication programming goes well beyond messages directed toward the primary audience of adolescent and adult men. For example, Table S2 presents an overview of a demand creation strategy for Nyanza Province, Kenya, produced by the USAID-funded program C-Change (managed by the Academy for Educational Development) in 2010. A comprehensive program functions at multiple levels, reaching multiple audiences in an effort to achieve multiple outcomes.

Perhaps most challenging, public health officials at all levels lack hard data on the cost of “convincing one individual to adopt one public health behavior,” and this is certainly the case for “triggering the decision” to seek MC services. As of 2006, there were only 35 studies in the published literature on the cost-effectiveness of behavior change communication for health interventions in low- and middle-income countries [20]. Not surprisingly, the reported costs differed markedly by type of health behavior. In terms of demand creation for VMMC, to our knowledge no studies have been published to date on attempts to evaluate the effectiveness of VMMC communication, let alone its cost-effectiveness (i.e., the cost for demand creation per VMMC performed).

Even when the “cost per unit of change” (that is, costs related to demand creation activities per VMMC performed) is estimated in one population or setting, this estimate would not be generalizable to other populations, given variations in the supply of and demand for VMMC services. In geographical areas with a high latent demand for VMMC (e.g., a community with many “early adopters”), it would be less costly to create demand for one VMMC than in an area with relatively low demand because of, for example, cultural norms that previously have not included MC, or where there is high pre-existing circumcision prevalence. In short, any estimate on the cost of demand creation in the scale-up of VMMC will be imprecise by definition. Yet the compelling need for cost estimates to assist in planning and budgeting justifies further pursuit of this question.

In sum, although the concept of a “standard package” for VMMC demand creation is desirable to planners, it is not realistic in practice. One can assemble a list of activities that serve as a widely used “menu” of the most common communication activities. Yet the allocation of costs to these different items will vary depending on a number of factors cited above. In fact, tailoring demand creation to the specific context increases its effectiveness and makes it more cost-efficient.

### Illustrative Case Study with Cost Data on Demand Creation for VMMC in Tanzania

To date, there has been very little systematic costing of demand creation activities. Although programs keep financial records of expenses for demand creation activities as part of their overall VMMC projects or programs, few have extracted these data for purposes of analyzing the distribution across different channels. One exception is the Jhpiego program in Tanzania. In this section, we present a description of the context for the VMMC program and the demand creation activities conducted in two regions—Iringa and Njombe—during an 8-wk period from June to August 2011.

#### Demand creation in the Iringa Region—*Dondosha mkonosweta!*

In February of 2011, authorities in the Iringa Region of Tanzania, with support from the USAID-funded Maternal and Child Health Integrated Program (MCHIP), through Jhpiego—an affiliate of Johns Hopkins University—began preparations for a June/July 2011 VMMC campaign designed to serve 20,000 clients in 8 wk at 21 sites. Concerned that the easy-to-reach clients had been served during the previous campaign and normal service delivery, the team endeavored to design a multi-channel communications initiative to launch and sustain the campaign. Focus group discussions were held to better understand male and female opinions about VMMC, garner their levels of knowledge about the purpose of VMMC, and gauge their knowledge of service locations. This formative assessment was used to design a communications campaign using the local slang term for the foreskin. *“Dondosha mkonosweta! Kitendo rahisi, sasa ni bure!”* (“Take off your sweater sleeve! Easy to do, now free!”) was tested and approved with the target audience and became the campaign slogan. Materials designed with the new slogan included print media, mass media, and text messaging.

#### Print media

Print media included the following: billboards targeting men and women placed in key markets and road stops; posters promoting the campaign and service sites (paper and plastic—for longer durability in the wet climate) (see Text S2); flyers advertising the campaign and service sites; pop-up banners to use at outreach activities, the launch event, and for the exteriors of service sites; and informational brochures for men, women, and adolescents and their guardians.

#### Mass media

Radio is the primary mass media in the Iringa Region. Therefore the campaign produced three radio spots (one targeting men, one targeting women, and one with general information) and aired them in heavy rotation (at least eight times per day) on a regional radio station and community stations in facility catchment areas. Broadcasts of these spots began one week before the campaign launch and ran until 2 wk before the end of the campaign. Additionally, radio talk shows featuring regional officials aired during the campaign period.

#### Text messaging

Print and mass media materials were used to promote a text messaging service, as described in the results from the key informant interviews above.

#### Community

The team again relied on HIV prevention partners (also funded by USAID) to promote VMMC through their scheduled interpersonal communication activities. These partners received print materials and training from MCHIP on VMMC messages. The campaign teams conducted outreach visits to community leaders and groups, primary and secondary schools, and churches in the facility catchment areas. An experiential media agency also promoted the campaign in facility communities through road shows, community events, and football games. An “emergency team” was designed to be quickly deployed to sites experiencing ebbs in client flow in order to create more demand.

#### Media campaign results

Although no formal evaluation has been completed of this demand creation activity, the June/July 2011 VMMC campaign in the Iringa Region served 31,046 clients, more than 150% of the intended number. The campaign reached more than 150,000 people during experiential media activities alone. The mix of print and mass media, coupled with community-based activities, helped bring clients in large numbers to the service delivery sites.

**Figure 1:**
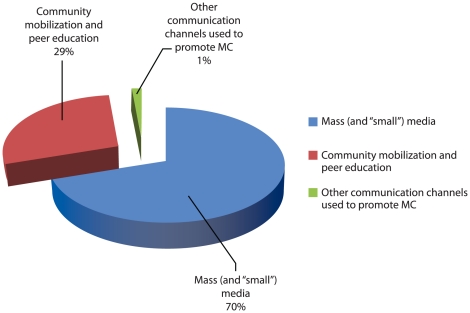
Percentage of budget for demand creation costs for Jhpiego VMMC campaigns in Iringa and Njombe Regions, Tanzania.

Jhpiego tracked the costs for demand creation activities in this campaign. The data in Table S3 indicate that the expenses for demand creation across channels came to US$266,950. As shown in Figure 1, 70% of the funding supported mass and small media, 29% was used for community mobilization, and 1% went to other channels. The final column in Table S3 presents a more detailed percentage breakdown by specific channel of communication. The largest demand creation expenses (40% of the total) were for the production of pamphlets, flyers, posters, and other supporting print materials for clients/parents. Following in second place was drama and street theater (27%). Production cost and rental space for billboards represented 24% of the total. Other categories of demand creation expense included the production and broadcast of materials for radio (5%), cell phone messaging (2%), honoraria/per diem payments for mobilizers and peer educators (1%), and training for mobilizers and peer educators (1%).

In presenting data on this program, we must warn against any interpretation that “one size fits all” in terms of demand creation. If one considers even a handful of the more active VMMC programs, this diversity becomes readily apparent. Orange Farm in South Africa represents one VMMC site with a community-wide rollout and a gradually expanding radius of influence; VMMC programs with different models are evolving in other parts of South Africa. The Kenya program has concentrated largely on a single province, Nyanza, but with high intensity of activity. Tanzania has focused its programmatic efforts on several provinces. Swaziland is unique, with an accelerated scale-up to reach a high percentage of males in a traditionally non-circumcising society. Thus, these data should be considered as descriptive of one program only: the demand creation efforts implemented by MCHIP in Tanzania.

## Discussion

The key informant interviews conducted in this study revealed a wide range of views on the elements of a standard package for VMMC demand creation, as reflected by the diversity of answers on resource allocation across channels. A first step toward estimating future costs would be to collect actual cost data to learn how countries have allocated their demand creation resources to date. Clearly, data should be collected from all organizations involved in VMMC demand creation in a given country to learn how countries allocate their demand creation resources between organizations that would likely play complementary roles.

Second, the primary channels for effective demand creation for VMMC include targeted radio and interpersonal communication or outreach, accompanied by print materials tailored for both men and women. Television, billboards, and other media play minor supporting roles in most VMMC scale-up settings. Depending on the costs charged by local phone companies, text messaging can represent a low-cost, promising strategy that deserves further exploration.

Third, these common elements do not lend themselves to a standardized package for demand creation that is applicable across countries. Rather, each country should determine the communication strategies and elements for successful demand creation, from which the costing estimates will derive. To complicate the matter further, different elements may be used in different parts of the program (e.g., billboards in urban but not rural areas). The mantra that communication programs must be tailored to the local needs of a specific country or regional context holds for VMMC.

Fourth, costing demand creation presents a greater methodological challenge than costing service delivery, in part because there is no straight line “dose–response” relationship (i.e., the delivery of a given amount of communication does not yield a predictable uptake in VMMC services). For example, it is possible to estimate the service delivery costs to provide 1,000 VMMC procedures in a given location with fair accuracy. However, one cannot predict with the same accuracy the increase in service uptake that will be generated by a given level of demand creation activity.

Fifth, it is unclear how demand creation costs may need to vary over time. One could argue that demand creation for VMMC services would require a large initial investment to create awareness, which then would be followed by smaller investments once a critical mass of knowledge has been created. Alternatively, one could argue that, in the early stages of scale-up, there may be latent demand for VMMC and that only over time, once this demand has been met, would there be a need for demand creation among those who need to be convinced of the value of VMMC.

This study has several limitations. First, the number of informants was small, hence the word “exploring” in the title of this article. Second, the key informant interviews did not include a wider group of participants such as government officials working in VMMC programs in the prioritized countries. These individuals are central to the strategic direction of demand generation programming and implementation in country. However, at the time of this data collection, they did not manage the budgets for demand creation activities, making it difficult for them to answer questions regarding the cost of different elements of the campaign. For this reason, the researchers selected persons known to manage large United States President's Emergency Plan for AIDS Relief–funded VMMC communication budgets. In future studies of this nature, it will be important to increase the number of key informant interviews, include a larger number of the 13 countries, and ensure the participation of a wider range of participants, including government officials responsible for the VMMC scale-up.

Based on these findings and caveats, our proposed approach requires country-specific analysis, with the data for each country aggregated to reach a total across the 13 countries working to scale up VMMC. The steps in the proposed approach are as follows. (1) Base the costing exercise on existing estimates of the number of adolescent and adult males who would need to be circumcised to achieve the saturation level in each country (see Box 1). (2) Identify the provinces or populations with low levels of MC prevalence to prioritize for VMMC programs. (3) Access the demand creation strategy for each country, working with local communication experts to develop such a strategy if one has not been developed and adapting elements from other programs with well-defined strategies (e.g., the Nyanza Province VMMC communication guide [21]). (4) Assess the current rate of VMMC scale-up, the level of coverage already reached, and the coverage objective, so as to estimate the additional work needed in a given country. (5) Collect data on the levels of expenditures for VMMC demand creation from relevant organizations for two consecutive years (see Text S3 for sample format). (6) Define a 5-y demand creation program to reach 80% of uncircumcised males, with specific outputs by region for each year, taking into consideration the urban/rural distribution of men to be reached, the degree of difficulty of reaching different populations, the number of local languages into which materials would need to be translated, and related factors. (7) Budget the expenses in these plans; compare existing levels of expenditure on demand creation to those elaborated in the plan and adjust as necessary in light of current levels of expenditure.

**Box 1.** Recommendations by C-Change (Academy for Educational Development; 2010) on the design of a demand creation program for Nyanza Province, Kenya.

**At the individual level:** Continue provider support and client education, with a focus on promoting VMMC within the context of broader HIV prevention.

Specific outcomes for this level of programming include:

- Circumcised men practice HIV prevention.
- Uncircumcised men go for VMMC, get tested, heal safely, champion VMMC, and then practice HIV prevention.
- Women encourage their partners to go for VMMC and are not exposed to added risk by the newly circumcised males.

**At the family, peer, and community level:** Mobilize the community to demand VMMC and incorporate VMMC within broader healthy social norms and attitudes relating to HIV prevention and gender.

Specific outcomes for this level of programming include:

- Families and friends of potential VMMC clients are informed and supportive of VMMC.
- Service providers communicate effectively about VMMC.
- Peer educators mobilize effectively around VMMC.

**At the environmental level:** Enhance political support for VMMC, engage key institutions (education, business) and constituency-based networks to support VMMC mobilization efforts, and improve VMMC media coverage.

Specific outcomes for this level of programming include:

- Role models support VMMC.
- The media report accurately about VMMC.
- Elders of the largest ethnic group in Nyanza Province, the Luo, are supportive of VMMC.
- VMMC is promoted by Kenyan churches.
- VMMC is supported at Kenyan workplaces.

This approach represents a significant data collection challenge, even for countries that have cost data available on VMMC activities. For those that have yet to begin the start-up in a major way, it may be necessary to use the costs of a “peer country” to impute cost estimates.

Three adjustments to such cost estimates are required. First, it is essential to budget the cost of designing a systematic strategy for demand creation, rather than simply estimating production and distribution costs. Most countries with strong demand creation efforts have benefited from external technical assistance for program design, and such costs must be figured into the calculations. Second, it is important to introduce an adjustment factor for start-up, on the assumption that a country's program must achieve some momentum before demand creation activities begin to increase service utilization. It stands to reason that certain portions of the population will be more likely to participate in the VMMC program from the outset and less likely to need encouragement from demand creation outreach. Third, it is important to estimate the added cost of communication programs intended for “harder-to-reach” populations, such as men over 25 y of age. Operational research on the costs of effective demand creation among these men in comparison to those 15–25 y of age could inform the field usefully.

In estimating costs, assumptions should be made explicit. Formative research and pretesting of materials should always form part of essential demand creation costs in a well-designed communication program. Although monitoring and evaluation of demand creation activities inform iterative adjustments to create and sustain strong programs, these costs should be tabulated as part of the overall monitoring and evaluation budget, rather than in the communication budget. Further, estimated costs for demand creation exclude costs related to client counseling. Although counseling contributes to confirming an individual decision to move forward with VMMC, it can be argued that by the time clients are in the clinic to receive that counseling, the demand already has been created.

We base this article on the premise that demand creation is essential for the scale-up of VMMC in eastern and southern Africa. This assumption stems from the fact that almost every major public health intervention designed to bring about behavior change (e.g., condom promotion to prevent HIV, use of bed nets to reduce malaria, adoption of contraception to space or limit births) has both supply and demand components. There is a growing literature on the effectiveness of communication campaigns in different areas of public health (e.g., meta-analysis performed by Snyder [19]). To date, we have no rigorous evaluation of demand creation for VMMC. Yet as programs advance, we anticipate that evaluation data will be forthcoming on the effectiveness of VMMC demand creation interventions on increasing VMMC uptake.

Finally, costing demand creation for VMMC assumes political will on the part of governments in the 13 countries to mobilize their own domestic resources and/or make use of available funds from international development partners to support VMMC scale-up, including effective demand creation programs. Where this is not the case and, even more markedly, where there is opposition to the promotion of VMMC, these estimates could be rendered useless rather than informing decision making on program planning.

Despite these challenges, we believe that the 13 priority countries in eastern and southern Africa can arrive at a reasonable estimate of the costs of demand creation for their VMMC services by following the steps outlined in this paper.

## Supporting Information

Table S1 **Types of communication channels that have been used to promote VMMC in eastern and southern Africa.**
(DOCX)

Table S2 **Estimated number of additional VMMCs needed to reach saturation levels in 13 eastern and southern African countries.** See also [22].
(DOCX)

Table S3 **Demand creation costs for the Jhpiego campaign in Iringa and Njombe Regions, June–August 2011.**
(DOCX)

Text S1 **Discussion guide for the key informant interviews on demand creation for male circumcision.**
(DOC)

Text S2 **Sample poster from print media portion of Iringa Region VMMC campaign.**
(TIFF)

Text S3 **Instructions for providing costs data for male circumcision demand creation.**
(DOC)

## Abbreviations

MC: male circumcision
MCHIP: Maternal and Child Health Integrated Program
SMS: short message service
UNAIDS: Joint United Nations Programme on HIV/AIDS
USAID: United States Agency for International Development
VMMC: voluntary medical male circumcision
