# Supplementary material for: Voluntary Medical Male Circumcision: A Qualitative Study Exploring the Challenges of Costing Demand Creation in Eastern and Southern Africa
Source: PLoS One. 2011 Nov 29;6(11):e27562. doi: 10.1371/journal.pone.0027562 (PMC3226625; doi:10.1371/journal.pone.0027562)
Supplement: Table S1 — Types of communication channels that have been used to promote VMMC in eastern and southern Africa. (DOCX) [file pone.0027562.s001.docx]

Table 1. Types of communication channels that have been used to promote MMC in Eastern and Southern Africa

| MASS AND SMALL MEDIA |
| --- |
| Radio:   - Radio spot - Radio coverage by local reporters (such as a news report about your site) - Radio call-in talk show - Other (radio) |
| Television:   - Television spot - Television coverage by local news reporters about the MMC service - Television call-in talk show - Other publicity (television) |
| Newspaper ad |
| Billboard |
| Posters:   - In clinics - In other public places |
| Pamphlet (or flyer):   - For MMC client - For spouse or partner of client - For general population (different from client or spouse pamphlets) |
| Video   - For prospective clients (to show in waiting room) - For general population |
| COMMUNITY LEVEL EVENTS |
| Visits/talks/mobilization in the following venues:   - Group meetings in the community - Schools - Factories, industries, mines, plantations - Military installations - Churches, mosques - Bar halls - Taxi stands, bus stops, motor bike stands - Prisons - Meetings with opinion leaders, influencers in the community - Van, truck, or other mobile vehicle that circulates in the community to promote MMC |
| Peer education activities (different from mobilization activities above)   - Satisfied clients |
| OTHER TYPES OF COMMUNICATION |
| - Cell phone messages re: MMC - Telephone hotline - Internet website for prospective clients - Song that promotes MMC - Dramas or plays about MMC (such as street theater) - Testimonials by a celebrity or public figure who has undergone MMC - Other |
