# Supplementary material for: Voluntary Medical Male Circumcision: A Qualitative Study Exploring the Challenges of Costing Demand Creation in Eastern and Southern Africa
Source: PLoS One. 2011 Nov 29;6(11):e27562. doi: 10.1371/journal.pone.0027562 (PMC3226625; doi:10.1371/journal.pone.0027562)
Supplement: Table S2 — Estimated number of additional VMMCs needed to reach saturation levels in 13 eastern and southern African countries. See also [22]. (DOCX) [file pone.0027562.s002.docx]

Table S2. Estimated number of additional MMCs needed to reach saturation levels in 13 Eastern and Southern African countries [21]

| Country | Population 15–49 years | Prevalence of MC at baseline | Additional MCs needed (#)  2011–2025 |
| --- | --- | --- | --- |
| Botswana | 1,077,230 | 10.20% | 487,658 |
| Lesotho | 1,055,461 | 0.00% | 544,165 |
| Malawi | 6,841,108 | 20.70% | 3,037,585 |
| Mozambique | 10,318,506 | 59.50% | 1,501,333 |
| Namibia | 1,086,239 | 21.00% | 477,910 |
| Kenya | 1,511,635 | 44.80% | 361,416 |
| Rwanda | 4,491,580 | 10.00% | 2,526,405 |
| South Africa | 26,836,874 | 44.70% | 5,944,785 |
| Swaziland | 589,680 | 8.20% | 267,946 |
| Tanzania | 19,630,363 | 66.80% | 1,971,888 |
| Uganda | 14,159,444 | 24.80% | 6,769,660 |
| Zambia | 6,844,545 | 10.80% | 3,495,351 |
| Zimbabwe | 6,187,130 | 10.30% | 2,680,409 |
