# Supplementary material for: Voluntary Medical Male Circumcision: A Qualitative Study Exploring the Challenges of Costing Demand Creation in Eastern and Southern Africa
Source: PLoS One. 2011 Nov 29;6(11):e27562. doi: 10.1371/journal.pone.0027562 (PMC3226625; doi:10.1371/journal.pone.0027562)
Supplement: Table S3 — Demand creation costs for the Jhpiego campaign in Iringa and Njombe Regions, June–August 2011. (DOCX) [file pone.0027562.s003.docx]

Table S3. Demand creation costs for the Jhpiego campaign in Iringa and Njombe, June-August 2011.

| **Communication activity** | **Amount in U.S. dollars** | **Percent of total for demand creation** |
| --- | --- | --- |
| **Applied research** | **0** | **0%** |
| Formative research for the purpose of designing communication materials for MC | 0 | (0%) |
| Pretesting of MC communication materials prior to production or dissemination | 0 | (0%) |
|  |  |  |
| **Mass and small media** | **186,154** | **70%** |
| Production and broadcast of materials for radio | 14,639 | (5%) |
| Production and broadcast of materials for television | 0 | (0%) |
| Production and rental of space: billboards | 63,679 | (24%) |
| Newspaper ads, commentary | 0 | (0%) |
| Production of pamphlets, flyers, and other print materials for clients/partners , posters, T-shirts | 107,836 | (40%) |
| Production & Distribution of posters | 0 | (0%) |
| Production of videos to promote MC | 0 | (0%) |
|  |  |  |
| **Community mobilization and peer education** | **76,624** | **29%** |
| Training for community mobilisers and peer educators, FGD and pre testing of brochures | 1,727 | (1%) |
| Salary, per diem, honorarium, other cash payments for mobilisers and peer educators | 0 | (0%) |
| (Per Diem District Govt Staff+ Vehicle fuel support.) | 2,569 | (1%) |
| Vehicle (purchase, rental. maintenance) used for MC promotion | 0 | (0%) |
| Drama, street theater (Experiential Media) | 72,328 | (27%) |
| Launching | 0 | (0%) |
|  |  |  |
| **Other communication channels used to promote MC** | **4,172** | **2%** |
| Hotlines | 0 | (0%) |
| Cell phone messaging | 4,172 | (2%) |
| Websites for prospective clients, partners | 0 | (0%) |
|  |  |  |
| **Total** | **266,950** | **100%** |
|  |  |  |
