# Supplementary material for: Voluntary Medical Male Circumcision: A Qualitative Study Exploring the Challenges of Costing Demand Creation in Eastern and Southern Africa
Source: PLoS One. 2011 Nov 29;6(11):e27562. doi: 10.1371/journal.pone.0027562 (PMC3226625; doi:10.1371/journal.pone.0027562)
Supplement: Text S1 — Discussion guide for the key informant interviews on demand creation for male circumcision. (DOC) [file pone.0027562.s004.doc]

Text S1 DISCUSSION GUIDE FOR THE KEY INFORMANT INTERVIEWS ON DEMAND CREATION FOR MALE CIRCUMCISION

Instructions to key informants: we will try to touch on all the questions below but realize that time may be a limiting factor. We’ll begin the call by reiterating the reason for needing this information, and then proceed with the following questions.

1. What are the elements of a standard package?
   1. Mass media channels
      1. What are the different types of media required? Which are essential and which are “nice but not essential”? (Examples: Television adverts necessary or a luxury? Is radio the key media approach? Would billboards be appropriate?)
      2. Is there any information on the relationship between mass media funding required and the number of people who will adopt the service? Is there any comparable intervention for which there are good data (e.g., social marketing of condoms, vasectomy, bed nets, etc.)?
      3. To what extent is mass media likely to be “stand alone” vs. “integrated with other media messages”?
   2. Local outreach
      1. What are the key elements of local outreach (e.g., posters, peer education, community outreach, etc.)?
      2. When entering new communities, how do you determine the extent of resources to spend on local outreach?
      3. Is local outreach typically sustained, increased, decreased over time?
      4. To what extent is local outreach likely to be “stand alone” (e.g., go get circumcised) vs. “integrated with other local outreach programs” (use condoms, go get tested and, if negative, go get circumcised)?
2. Costing of communication/demand creation
   1. Have any of the country programs already costed MC communication? If so, can they provide their budgets?
   2. What would you consider to be an ideal resource allocation for communication for MC (e.g., 75% radio, 15% television, 10% billboards)?
   3. Is there information that we could obtain on media costs by country (e.g., differences of radio airtimes among countries)?
   4. Other than the cost of airtime, what are the factors that are likely to influence the cost of demand creation (e.g., knowledge of MMC, general acceptance of MMC, # of men to be circumcised, etc.)?
   5. How will resource allocation need to change with time (e.g., large initial messages that decline over time or small focused messages in the beginning and a larger “splash” once the early adopters have been reached)?
