# Supplementary figures and images for: Voluntary Medical Male Circumcision: A Qualitative Study Exploring the Challenges of Costing Demand Creation in Eastern and Southern Africa
Source: PLoS One. 2011 Nov 29;6(11):e27562. doi: 10.1371/journal.pone.0027562 (PMC3226625; doi:10.1371/journal.pone.0027562)

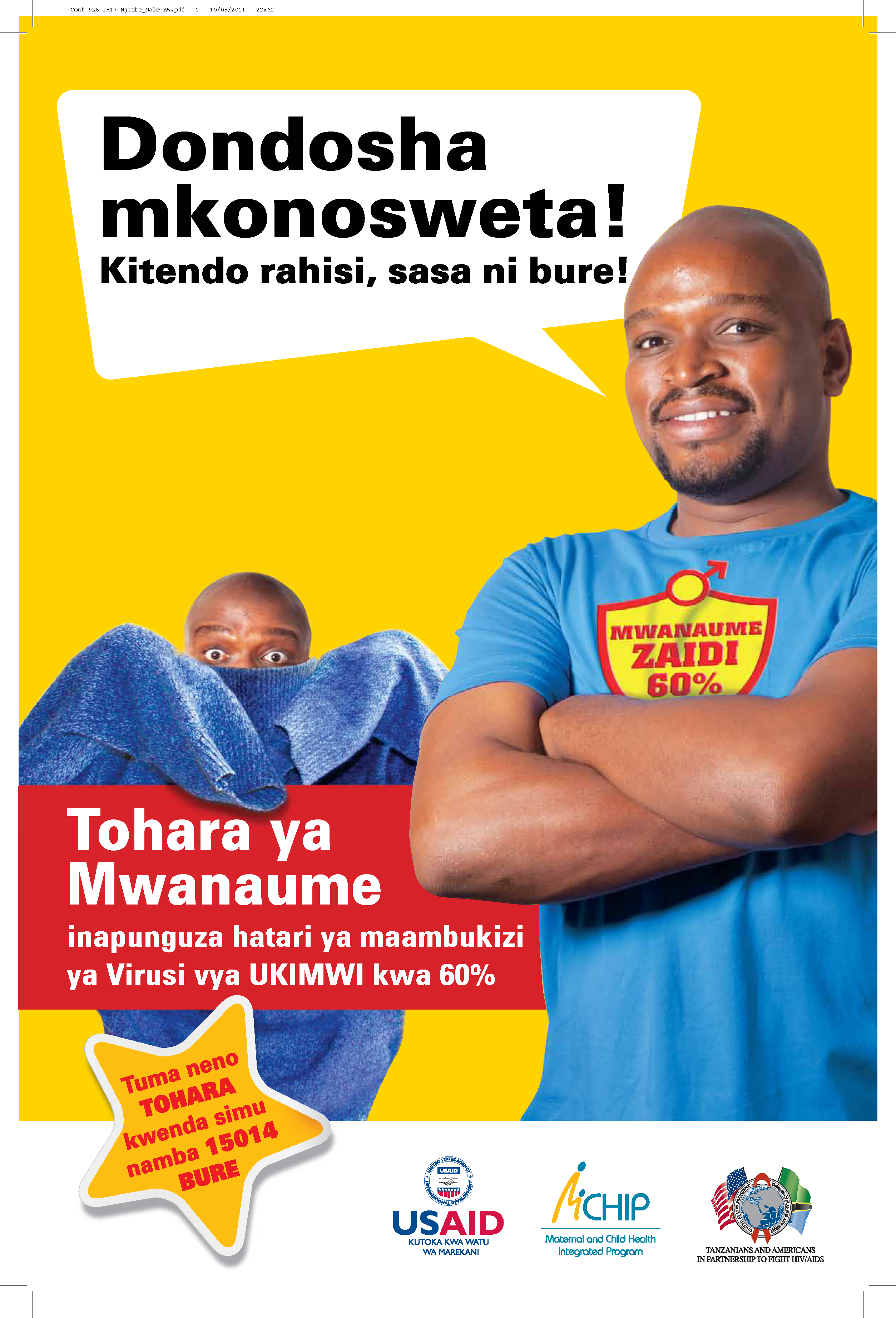

Supplement: Text S2 — Sample poster from print media portion of Iringa Region VMMC campaign. (TIFF) [file pone.0027562.s005.tiff]
