# Supplementary material for: Voluntary Medical Male Circumcision: A Qualitative Study Exploring the Challenges of Costing Demand Creation in Eastern and Southern Africa
Source: PLoS One. 2011 Nov 29;6(11):e27562. doi: 10.1371/journal.pone.0027562 (PMC3226625; doi:10.1371/journal.pone.0027562)
Supplement: Text S3 — Instructions for providing costs data for male circumcision demand creation. (DOC) [file pone.0027562.s006.doc]

TEXT S3 INSTRUCTIONS FOR PROVIDING COST DATA ON MALE CIRCUMCISION DEMAND CREATION

Please provide information on the approximate costs (in U.S. dollars) of the following activities for medical male circumcision (MMC) demand creation in your country for FY 2010 and 2011.

Person completing this form: _____________________________________________

Position within organization: _____________________________________________

Name of organization: __________________________________________________

Country: _____________________________________________________________

|  | FY 2011:  Oct. 1, 2010–Sept. 30, 2011  (list costs in U.S. dollars) | FY 2012:  Oct. 1, 2010–Sept. 30, 2012 (list costs in U.S. dollars) |
| --- | --- | --- |
| Salaries and fringe benefits for staff dedicated to MMC demand creation (note: if a full staff member works 50% on MMC demand creation, list the 50%): | $ | $ |
| - Salary of staff working on MMC demand creation | $ | $ |
| Applied research: | $ | $ |
| - Formative research for the purpose of designing communication materials for MMC |  |  |
| - Pretesting of MMC communication materials prior to production or dissemination |  |  |
| Mass (and “small”) media: | $ | $ |
| - Production and broadcast of materials for radio |  |  |
| - Production and broadcast of materials for television |  |  |
| - Production and rental of space: billboards |  |  |
| - Newspaper ads, commentary |  |  |
| - Production of pamphlets, flyers, and other print materials for clients/partners |  |  |
| - Production of posters |  |  |
| - Production of videos to promote MMC |  |  |
|  |  |  |
| Community mobilization and peer education: |  |  |
| - Training for community mobilizers and peer educators |  |  |
| - Salary, per diem, honorarium, other cash payments for mobilizers and peer educators |  |  |
| - Vehicle (purchase, rental. maintenance) used for MMC promotion |  |  |
| - Drama, street theater |  |  |
|  |  |  |
| Other communication channels used to promote MMC: |  |  |
| - Hotlines |  |  |
| - Cell phone messaging |  |  |
| - Websites for prospective clients, partners |  |  |
|  |  |  |
| Other: specify |  |  |
|  |  |  |
|  |  |  |

How many staff do you have dedicated to MMC demand creation (including supervisors)?

- Full-time staff: _____
- Part-time staff: _____
- Volunteers: _____

Remarks: _____________________________________________________________________

____________________________________________________________________________

____________________________________________________________________________

____________________________________________________________________________

Notes for completing this exercise:

1. Provide all costs in U.S. dollars.
2. Prorate items that are partially but not 100% dedicated to MMC demand creation. For example, if a full-time staff member works 50% on MMC demand creation, list only 50% of salary/fringe benefits for that person. Similarly, if the work week has five days, and a vehicle is used for demand creation for only one of those five days, prorate this cost as 20% of the total vehicle purchase or rental.
3. Regarding staff, list the salaries and fringe benefits for all staff whose primary responsibility relates to MMC demand creation, including staff who supervise such workers (e.g., community mobilizers). For the purpose of this exercise, do NOT include staff members who provide counseling to those intending to have the MMC operation or counselors for HIV testing services.
4. List “$0” for any activity or item not relevant to your program.
5. Try to give the most precise estimates possible. However, we realize that it may be necessary to approximate when exact estimates are not available; this is acceptable.
6. Provide any additional notes in the Remarks section or on a separate piece of paper.

Thanks in advance for your assistance on this important data collection exercise.
